# On the Move or Barely Moving? Age-Related Changes in Physical Activity, Sedentary, and Sleep Behaviors by Weekday/Weekend Following Pandemic Control Policies

**DOI:** 10.3390/ijerph19010286

**Published:** 2021-12-28

**Authors:** Ann Pulling Kuhn, Alysse J. Kowalski, Yan Wang, Rachel Deitch, Helina Selam, Zahra Rahmaty, Maureen M. Black, Erin R. Hager

**Affiliations:** 1 Department of Pediatrics, School of Medicine, University of Maryland, Baltimore, MD 21201, USA; apullingkuhn@som.umaryland.edu (A.P.K.); akowalski@som.umaryland.edu (A.J.K.); rdeitch@som.umaryland.edu (R.D.); hselam@som.umaryland.edu (H.S.); mblack@som.umaryland.edu (M.M.B.); 2 Department of Prevention and Community Health, Milken Institute School of Public Health, George Washington University, Washington, DC 20052, USA; yanwang20@email.gwu.edu; 3 Department of Biology and Medicine, Institut Universitaire de Formation et de Recherche en Soins, Lausanne University Hospital, University of Lausanne, IUFRS Bureau 169—SV-A Secteur Vennes—Rte de la Corniche 10, CH-1010 Lausanne, Switzerland; Zahra.Rahmaty@unil.ch; 4 RTI International, Research Triangle Park, Durham, NC 27709, USA; 5 Department of Epidemiology and Public Health, University of Maryland School of Medicine, Baltimore, MD 21201, USA

**Keywords:** COVID-19, physical activity, children

## Abstract

This study examined pre-pandemic (2017-early March 2020) to early-pandemic (Spring 2020) changes in moderate-to-vigorous PA (MVPA), light PA (LPA), and sedentary behavior/sleep (SS), by weekday/weekend, and age (preschool, elementary, middle school). We re-enrolled children from two pre-pandemic obesity prevention trials and examined differences in accelerometer-measured PA from pre-pandemic to early-pandemic across age groups using linear mixed models. Children (*n* = 75) were 51% multiple race/ethnicities, 29% preschool, 28% elementary, 43% middle school, 65% suburban, 21% rural, and 13% urban. Pre-pandemic to early-pandemic changes in weekday MVPA (*p* = 0.006), LPA (*p* = 0.018), and SS (*p* = 0.003) differed by age. On weekdays, middle schoolers’ MVPA decreased 15.36 min/day (*p* = 0.002) and SS increased 94.36 min/day (*p* < 0.001) with non-significant changes among preschoolers and elementary schoolers. Compared to elementary schoolers, middle schoolers’ changes in weekday MVPA (*b* = −16.34, *p* = 0.036) and SS (*b* = 63.28, *p* = 0.039) significantly differed. Declines in weekday MVPA and increases in SS among middle schoolers suggest that, compared with younger children, middle schoolers are dependent on school and recreational facilities for PA, and in their absence engage in more sedentary activities and sleep.

## 1. Introduction

Spring 2020 pandemic-control policies in a mid-Atlantic state in the U.S. included school and recreation facility closures that began 12 March 2020. From this time through the end of the school year, school-age children and adolescents attended virtual school from home and preschoolers were home without a virtual option. These restrictions reduced children’s access to school-based opportunities for physical activity (PA) including physical education, recess, active transportation to and from school, or other PA opportunities during the school day. Based on U.S. pre-pandemic data, 76% of children do not meet the nationally recommended 60 min of daily moderate to vigorous PA (MVPA) [1]. Given the harm of high sedentary behaviors [2] and benefits of PA on children’s fitness and motor competence development [3], the lack of instructions and opportunities to engage in PA due to pandemic-related restrictions could have deleterious effects on children’s fitness and motor competence during the pandemic, with lasting consequences into the future.

Pandemic-related PA changes measured through surveys have shown inconsistent results. One review, based primarily on caregiver report, found that preschool and school-age children’s time in MVPA decreased due to pandemic-related social isolation [4]. However, a study from Ireland among adolescent girls found no significant change in youth-reported PA [5]. In contrast, studies using accelerometry have shown declines in PA related to the pandemic. A study from Spain with four to six-year-old preschoolers found mean pandemic-related PA declines of 43 min/day and sedentary behavior increases of 50 min/day [6]. Likewise, a study from Holland among children aged 7–12 years found mean pandemic-related increases of 45 min/day in sedentary time and decreases from 64% to 20% in the percentage of children who met the recommended PA levels of 60 min/day [7]. A study from the U.S. examined 231 children aged 7–12 years and found that light PA and MVPA decreased by 69 and 8 min respectively, while sedentary behavior increased by 79 min and sleep increased by 17 min [8].

Most studies of pandemic-related changes in PA have not examined weekday versus weekend differences. The ‘structured days hypothesis’ suggests that days with intentional, pre-planned activities outside the home, with defined start/end times, support health-promoting behaviors since time filled with favorable activities cannot be filled with unfavorable activities, based on the filled-time perspective [9]. Unstructured days may allow children to choose sedentary pursuits over PA in the absence of pre-planned opportunities, and there is less regulation/restriction which may allow for increased sedentary/screen time. This hypothesis can be applied to the onset of the pandemic when children experienced a series of unstructured days (similar to weekend days). Given this, we sought to examine pre- to early-pandemic differences in PA separately for weekdays and weekends, hypothesizing a greater change in PA for weekdays versus weekends.

Several factors may be associated with changes in PA following the onset of pandemic control polices for children. Prior to the pandemic, age differences in activity were consistently documented such that preschool-aged children were more physically active outside of school than older children [10] and PA declines were commonly observed during the transition from childhood to adolescence [11]. Based on data from over 24,000 children aged 5 to 18 years in the International Children’s Accelerometry Database, objectively-measured vigorous physical activity declined 6.9% for every year of age, representing a reduction of 7.8 min per year [12]. These studies suggest that long term school closures could lead to a larger gap in PA by age. Other demographic factors, including sex, race, socioeconomic status, weight status, and geographic locale (i.e., urban, suburban, rural) have been associated with children’s PA prior to the pandemic [13,14,15] and may be related to pandemic-related changes in PA. Furthermore, pre-pandemic PA has been shown to be related to PA during the pandemic among adults [16], which warrants investigating this factor in children.

The purpose of this study was to examine pre-pandemic (2017–early March 2020) to early-pandemic (5/2020–7/2020) changes in MVPA, light intensity PA (LPA), and sedentary behavior/sleep (SS), by weekday/weekend and age (preschool, elementary, and middle school). Guided by the structured days hypothesis that structured school-based activities support PA, with children more active on weekdays than weekends [9,17], we hypothesized weekday PA would decrease and weekend PA would not change following implementation of pandemic-control policies. We also explored if MVPA, LPA, and SS changes differed by age and pre-pandemic MVPA.

## 2. Materials and Methods

### 2.1. Participants 

Children from two statewide childhood obesity prevention intervention trials, Creating Healthy Habits Among Maryland Preschoolers (CHAMP) and Wellness Champions for Change (WCC), were recruited and re-enrolled in the COVID-19 Family Study (an observational cohort of children and their families followed during the pandemic) [18,19]. Both trials aimed to improve child diet and PA and enrolled three cohorts over three academic years (2017, 2018, 2019) with pre-pandemic data collected from 10/2017 to early 3/2020, prior to pandemic-related closures. The CHAMP and WCC studies took place in 54 childcare centers and 33 schools (18 elementary and 15 middle) serving diverse communities, in thirteen counties across Maryland. Childcare centers were eligible if they accepted childcare vouchers, participated in the Child and Adult Care Food Assistance Program, or cost less than $300/week per child. Elementary and middle schools were eligible if >40% of the student body was eligible for free or reduced-price school meals. Children with pre-pandemic survey data were eligible to participate in the COVID-19 study. Protocols for CHAMP (HP-00065933), WCC (HP-00067626) and COVID-19 (HP-00090906) were approved by the University of Maryland School of Medicine Institutional Review Board. Caregivers provided written informed consent for the original studies and re-consented for the COVID-19 study. Children provided assent.

**Figure 1:**
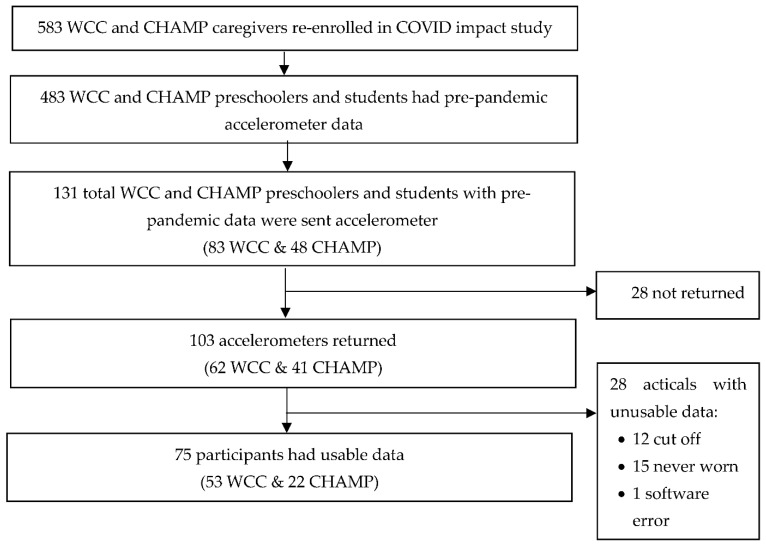
Participant flow chart.

Of 583 children who re-enrolled in the COVID-19 study, 483 children had pre-pandemic survey and accelerometer data. We used stratified randomization by age, gender, and school locale to identify 131 children to participate in the accelerometer sub-study. Of the 131 mailed accelerometers, 103 were returned, and 75 had at least one 24-h day of valid PA data and were included in the analysis (see Figure 1). Participant (*n* = 75) pre-pandemic characteristics were 63% female, 49% white, 33% black/African American, 15% mixed race, 1% Asian, 39% overweight/obese, 65% suburban, 21% rural, and 13% urban. In Spring 2020, 22 were preschoolers (3–5 years), 21 elementary schoolers (6–10 years), and 32 middle schoolers (11–14 years).

### 2.2. Data Collection and Measures

#### 2.2.1. Physical Activity

Physical activity data were collected at two timepoints. Baseline data were collected from 10/2017 to early 3/2020, prior to pandemic-related closures (referred to as “pre-pandemic” henceforth). Follow-up data were collected from 5/2020–7/2020 during the first wave of the pandemic (“early-pandemic” henceforth).

At pre-pandemic, study staff placed the accelerometers on the children’s non-dominant ankle. During early-pandemic, we e-mailed a video that illustrated the proper placement of the accelerometer. We mailed an accelerometer (Actical, formerly Minimitter Co., Philips Respironics, Bend, OR, USA), instructions to wear the accelerometer for seven days without removal, and a pre-paid return envelope to caregivers who placed the accelerometers on their child’s non-dominant ankle and returned the accelerometers to study staff. Data, downloaded with Actical software (version 2.12) (Philips Respironics, Bend, OR, USA), included average minutes of MVPA, LPA, and SS using thresholds validated among adolescents [20] and toddlers [21], which have been previously used with preschool-aged children [22].

#### 2.2.2. Covariates

Caregivers reported the children’s demographics via an online or paper survey at pre-pandemic. Variables included gender, age, race/ethnicity, and socioeconomic status (SES; determined by federal poverty levels). We collected school locale data (i.e., rural, suburban, urban) from the National Center for Education Statistics or school websites. Children’s BMI (kg/m^2^) was calculated from height and weight measurements collected by research assistants at baseline. BMI-for-age percentiles were further categorized into categories of weight status (underweight: <5th percentile; healthy weight: 5th to >85th percentile; overweight: 85th to >95th percentile; obese: ≥95th percentile) [23].

### 2.3. Statistical Analysis

For descriptive statistics, means and standard deviations were calculated for continuous variables and frequencies were calculated for categorical variables. Independent samples *t*-tests and chi-square tests tested differences between participants with usable data (*n* = 75) and participants who were sent an accelerometer, but did not have usable data (*n* = 56). We used separate linear mixed models with a random intercept to account for repeated measures to explore pre-pandemic to early-pandemic changes in weekday and weekend MVPA, LPA, and SS. In each model we included interaction terms between time point (pre-pandemic/early pandemic) and age category, and pre-pandemic minutes of MVPA to examine if changes in MVPA, LPA, or SS differed by these characteristics. Models were adjusted for sex, race, weight status, locale, SES, intervention status (whether participants were in the control or intervention group in the original trial), season of accelerometry measurement (i.e., defined as same or different season compared to season at pre-pandemic data collection), and follow-up time (time in years between pre-pandemic and early pandemic measurements). Data were analyzed using SPSS version 27 (IBM Corp, Armonk, NY, USA), Stata version 17 (Stata Corp, College Station, TX, USA), and R version 4.0.3. (R Core Team, Vienna, Austria).

## 3. Results

Based on independent samples *t*-tests and chi-square tests, excluded participants (*n* = 56) were more likely to be male (*p* = 0.007), but there were no other significant differences between included (*n* = 75) and excluded (*n* = 56) participants based on race (*p* = 0.179), age (*p* = 0.450), locale (*p* = 0.194), weight status (*p* = 0.410), or SES (*p* = 0.058). Weekday and weekend MVPA, LPA, and SS had skewness and kurtosis scores within ±1, indicating that they did not deviate from normal distribution [24].

**Figure 2:**
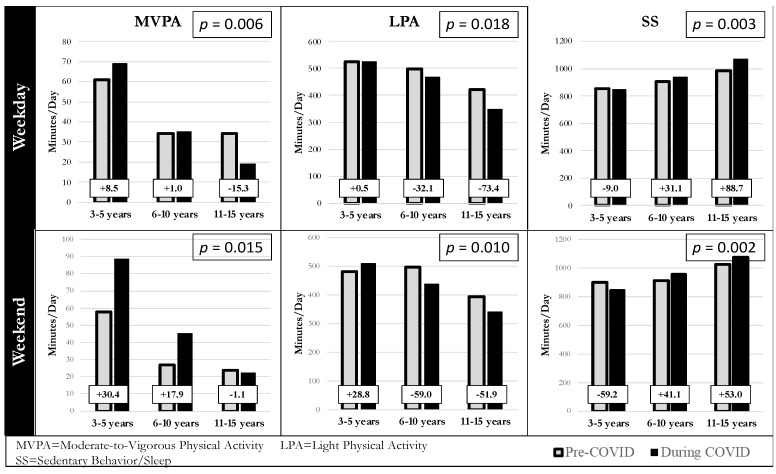
Raw mean change in weekday and weekend accelerometer-measured physical activity (*n* = 75) from pre-pandemic (2017–2019) to early-pandemic (Spring 2020) by age group (preschool [*n* = 22]; elementary [*n* = 21]; middle [*n* = 32]), as determined by age at pre-pandemic. *p*-values indicate significant age-by-time interactions from linear mixed models.

Figure 2 shows changes from pre-pandemic to early-pandemic in raw minutes of weekday and weekend MVPA, LPA, and SS by age group. For weekday MVPA, preschoolers increased by 8.5 min per day, elementary schoolers increased by 1 min per day, and middle schoolers decreased by 15 min per day. For light PA, preschoolers showed little change, while elementary and middle schoolers decreased by 32 and 73 min respectively. Finally, preschoolers decreased by 9 min per day in SS, while elementary and middle schoolers increased by 31 and 88 min respectively. For weekend MVPA, both preschoolers and elementary schoolers increased by 30 and 17 min per day respectively, while middle schoolers showed a slight decrease. For light PA, preschoolers increased by 28 min per day, while elementary and middle schoolers both decreased by 59 and 51 min per day respectively. Finally, for SS, preschoolers decreased by 59 min per day while elementary and middle schoolers increased by 41 and 53 min per day.

### 3.1. Modeled Changes in Weekday MVPA, LPA, and SS

Table 1 shows results of the linear mixed models for weekday MVPA, LPA, and SS. A significant age-by-time interaction for weekday MVPA (*p* = 0.006), LPA (*p* = 0.018), and SS (*p* = 0.003) demonstrated that changes differed by age. Middle schoolers decreased 15.36 min/day in MVPA (*p* = 0.002), decreased 79.05 min/day in LPA (*p* < 0.001), and increased 94.36 min/day in SS (*p* < 0.001). Compared to elementary schoolers, middle schoolers’ changes in weekday MVPA (*b* = −16.34, *p* = 0.036) and SS (*b* = 63.28, *p* = 0.039) significantly differed. Changes in MVPA, LPA, and SS were nonsignificant among preschoolers and elementary schoolers, as shown in Table 1.

### 3.2. Modeled Changes in Weekend MVPA, LPA, and SS

Table 2 shows results of the linear mixed models for weekend MVPA, LPA, and SS. A significant age-by-time interaction for weekend MVPA (*p* = 0.015), LPA (*p* = 0.010), and SS (*p* = 0.002) demonstrated that changes differed by age. Preschoolers increased 18.11 min/day in MVPA (*p* = 0.030) and decreased 58.63 min/day in SS *(p* = 0.007). Elementary schoolers increased 30.08 min/day in MVPA (*p* = 0.007) and decreased 57.47 min/day in LPA (*p* = 0.008). Middle schoolers decreased 35.84 min/day in LPA (*p* = *0*.050). Compared to elementary schoolers, changes in preschoolers’ LPA (b = 85.96, *p* = 0.004) and SS (b = −97.54, *p* = 0.004) significantly differed.

There were no moderating effects of baseline MVPA on any outcomes.

## 4. Discussion

This study found that pre-pandemic to early-pandemic changes in children’s PA behaviors differed by age groups. Hypothesized declines in weekday MVPA and increases in weekday SS following pandemic-control policies occurred among middle schoolers, but not preschoolers or elementary schoolers.

Different responses to pandemic-control policies may be related to age differences in motor competencies and structure. Middle schoolers’ declines in weekday MVPA and increases in SS were significantly different from elementary schoolers, suggesting that they were more dependent on schools and recreational facilities for structured and organized PA opportunities (e.g., physical education, sports) than elementary schoolers. In contrast, preschoolers’ PA often includes unstructured activities, with relatively few opportunities for organized PA [25]. Thus, preschoolers and school-age children responded differently in the absence of structured school days, making the ‘structured days hypothesis’ more applicable to elementary and middle school children [9]. Furthermore, weekday LPA significantly decreased among middle schoolers and weekend LPA significantly decreased among elementary and middle schoolers. Since LPA may be important for students’ academic outcomes [26], future research should seek to improve elementary and middle school students’ LPA when not in school settings. Alternatively, preschoolers’ LPA changes were not significant, but their weekday LPA remained about the same, which is in line with another study that found no change in preschoolers’ LPA from pre- to early-pandemic [27].

The age-related pattern of change was similar for weekends, with preschoolers decreasing in SS, while older children increased. Since sedentary behavior is negatively associated with motor competence, particularly among elementary schoolers [2], it is important to ensure increases in SS are buffered against so that children remain active as they mature. Perhaps elementary and middle schoolers were sleeping more during the pandemic, as has been reported in at least one study [8]. Another study from Germany also found that the pandemic had stronger negative effects on adolescents’ PA than younger children [28]. Furthermore, declines in PA and increases in SS could be due to the well-documented changes in PA as children get older [10]. Contrary to previous studies [16,29], pre-pandemic PA was unrelated to PA changes, suggesting that children of all PA levels experienced early-pandemic-related changes.

A strength of this study is the use of objective measures to measure changes in PA from pre-pandemic to early-pandemic. This study was limited by the small sample size, which may limit generalizability; however, the subset was identified by pre-determined criteria to maximize demographic variability by age, sex, and locale. Furthermore, given the small sample size, the results must be interpreted with caution since adjusting for many factors can result in a loss of precision. Future research should examine changes in PA before to after the pandemic with a larger sample size. Additionally, the use of 24-h accelerometry led to the merging of sedentary behavior and sleep since we are not aware of an algorithm to extrapolate sleep from sedentary time for these age groups or device. A recent study was able to differentiate between sedentary behavior and sleep, finding that both sedentary behavior and sleep increased during the early pandemic (compared to pre-pandemic) among elementary school-aged children [8]. Additional research is needed to specifically understand the impact of pandemic control policies on weekday/weekend patterns of sleep, including sleep onset, duration, quality, etc. in addition to sedentary behaviors among children of different ages.

## 5. Conclusions

Overall, this study showed that structured activities offered by schools promote PA and may serve as protection against obesogenic behaviors such as the sedentariness we found, especially among middle schoolers [9]. As pandemic control policies evolve, efforts are needed to address declines in MVPA, (e.g., providing at-home family PA opportunities) particularly among middle schoolers, as PA patterns established in childhood are likely to continue into adulthood [30]. Findings confirm previous studies of children’s objectively measured PA during the COVID-19 pandemic [6,7] and highlight the well-documented differences in PA during structured and unstructured times and by age during non-pandemic conditions [9]. Future COVID-19 PA research should consider PA patterns by weekday/weekend and child age as these were important factors among children in this study.

## Figures and Tables

**Table 1:** Results of mixed model analyses for weekday MVPA, LPA, and SS (*n* = 74).

	Model #1: Weekday MVPA		Model #2: Weekday LPA		Model #3: Weekday SS	
Estimated Change in PA over Time by Age Group	M (SE)	*p*-Value	M (SE)	*p*-Value	M (SE)	*p*-Value
Elementary schoolers	0.98 (5.74)	0.863	−32.07 (20.93)	0.130	31.08 (22.62)	0.173
Preschoolers	8.53 (5.61)	0.128	0.47 (20.45)	0.981	−9.00 (22.09)	0.683
Middle schoolers	−15.36 (5.03)	0.002 **	−79.05 (18.37)	0.001 **	94.36 (19.87)	0.001 **
**Estimated Difference in Change in PA over Time (Ref: Elementary Schoolers)**	***b***	***p*-Value**	***b***	***p*-Value**	***b***	***p*-Value**
Preschoolers	7.55	0.350	32.54	0.270	−40.09	0.209
Middle schoolers	−16.34	0.036 *	−46.98	0.096	63.28	0.039 *

Note. Weekday MVPA, LPA, and SS as dependent continuous variables. * *p* < 0.05, ** *p* < 0.01. Adjusted for gender, race, SES, weight status, locale, intervention status, time since pre-pandemic measurement, and season (i.e., defined as same or different season compared to season at pre-pandemic data collection). Age groups: 3–5 years (*n* = 22); 6–10 years (*n* = 21); 11–15 years (*n* = 32). Means of changes in PA from pre-pandemic to early-pandemic are estimated based on models.

**Table 2:** Results of mixed model analyses for weekend MVPA, LPA, and SS (*n* = 68).

	Model #4: Weekend MVPA		Model #5: Weekend LPA		Model #6: Weekend SS	
Estimated Change in PA over Time by Age Group	M (SE)	*p*-Value	M (SE)	*p*-Value	M (SE)	*p*-Value
Elementary schoolers	30.08 (7.63)	0.007 **	−57.47 (21.57)	0.008 **	38.91 (23.85)	0.103
Preschoolers	18.11 (8.35)	0.030 *	28.49 (19.70)	0.148	−58.63 (21.77)	0.007 **
Middle schoolers	−0.69 (7.08)	0.923	−35.84 (18.29)	0.050 *	36.39 (20.22)	0.071
**Estimated Difference in Change in PA over Time (Ref: Elementary Schoolers)**	***b***	***p*-Value**	***b***	***p*-Value**	***b***	***p*-Value**
Preschoolers	11.97	0.294	85.96	0.004 **	−97.54	0.004 **
Middle schoolers	−18.80	0.090	21.64	0.446	−2.52	0.081

Note. Weekend MVPA, LPA, and SS as dependent continuous variables. * *p* < 0.05, ** *p* < 0.01. Adjusted for gender, race, SES, weight status, locale, intervention status, time since pre-pandemic measurement, and season (i.e., defined as same or different season compared to season at pre-pandemic data collection). Age groups: 3–5 years (*n* = 22); 6–10 years (*n* = 21); 11–15 years (*n* = 32). Means of changes in PA from pre-pandemic to early-pandemic are estimated based on models.

## Data Availability

The datasets used and/or analyzed during the current study are available from the corresponding author on reasonable request.

## References

[1] The Child & Adolescent Health Measurement Initiative (2016). 2016 National Survey of Children’s Health.

[2] Santos G.D., Guerra P.H., Milani S.A., Santos A.B.D., Cattuzzo M.T., Ré A.H.N. (2021). Sedentary behavior and motor competence in children and adolescents: A review. Rev. Saúde Pública.

[3] King-Dowling S., Proudfoot N.A., Cairney J., Timmons B.W. (2020). Motor Competence, Physical Activity, and Fitness across Early Childhood. Med. Sci. Sports Exerc..

[4] López-Bueno R., López-Sánchez G.F., Casajús J.A., Calatayud J., Tully M.A., Smith L. (2020). Potential health-related behaviors for pre-school and school-aged children during COVID-19 lockdown: A narrative review. Prev. Med..

[5] O’Kane S.M., Lahart I.M., Gallagher A.M., Carlin A., Faulkner M., Jago R., Murphy M.H. (2021). Changes in Physical Activity, Sleep, Mental Health, and Social Media Use During COVID-19 Lockdown Among Adolescent Girls: A Mixed-Methods Study. J. Phys. Act. Health.

[6] Alonso-Martínez A.M., Ramírez-Vélez R., García-Alonso Y., Izquierdo M., García-Hermoso A. (2021). Physical Activity, Sedentary Behavior, Sleep and Self-Regulation in Spanish Preschoolers during the COVID-19 Lockdown. Int. J. Environ. Res. Public Health.

[7] Ten Velde G., Lubrecht J., Arayess L., van Loo C., Hesselink M., Reijnders D., Vreugdenhil A. (2021). Physical activity behaviour and screen time in Dutch children during the COVID-19 pandemic: Pre-, during- and post-school closures. Pediatr. Obes..

[8] Burkart S., Parker H., Weaver R.G., Beets M.W., Jones A., Adams E.L., Chaput J.P., Armstrong B. (2022). Impact of the COVID-19 pandemic on elementary schoolers’physical activity, sleep, screen time and diet: A quasi-experimental interrupted time series study. Pediatr. Obes..

[9] Brazendale K., Beets M.W., Weaver R.G., Pate R.R., Turner-McGrievy G.M., Kaczynski A.T., Chandler J.L., Bohnert A., Von Hippel P.T. (2017). Understanding differences between summer vs. school obesogenic behaviors of children: The structured days hypothesis. Int. J. Behav. Nutr. Phys. Act..

[10] De Meester F., Van Dyck D., De Bourdeaudhuij I., Deforche B., Cardon G. (2014). Changes in physical activity during the transition from primary to secondary school in Belgian children: What is the role of the school environment?. BMC Public Health.

[11] Armstrong N., Welsman J.R. (2006). The Physical Activity Patterns of European Youth with Reference to Methods of Assessment. Sports Med..

[12] Corder K., Sharp S., Atkin A., Andersen L.B., Cardon G., Page A., Davey R., Grøntved A., Hallal P.C., Janz K.F. (2016). Age-related patterns of vigorous-intensity physical activity in youth: The International Children’s Accelerometry Database. Prev. Med. Rep..

[13] Sterdt E., Liersch S., Walter U. (2013). Correlates of physical activity of children and adolescents: A systematic review of reviews. Health Educ. J..

[14] Prentice-Dunn H., Prentice-Dunn S. (2012). Physical activity, sedentary behavior, and childhood obesity: A review of cross-sectional studies. Psychol. Health Med..

[15] Euler R., Jimenez E.Y., Sanders S., Kuhlemeier A., Van Horn M.L., Cohen D., Gonzales-Pacheco D., Kong A.S. (2019). Rural–Urban Differences in Baseline Dietary Intake and Physical Activity Levels of Adolescents. Prev. Chronic Dis..

[16] Teran-Escobar C., Forestier C., Ginoux C., Isoard-Gautheur S., Sarrazin P. (2021). Individual, Sociodemographic, and Environmental Factors Related to Physical Activity During the Spring 2020 COVID-19 Lockdown. Frontiers in Psychology. Front. Psychol..

[17] Brooke H., Corder K., Atkin A.J., van Sluijs E. (2014). A Systematic Literature Review with Meta-Analyses of Within- and Between-Day Differences in Objectively Measured Physical Activity in School-Aged Children. Sports Med..

[18] Lane H.G., Deitch R., Wang Y., Black M.M., Dunton G.F., Aldoory L., Turner L., Parker E.A., Henley S.C., Saksvig B. (2018). “Wellness Champions for Change,” a multi-level intervention to improve school-level implementation of local wellness policies: Study protocol for a cluster randomized trial. Contemp. Clin. Trials.

[19] Armstrong B., Trude A.C., Johnson C., Castelo R.J., Zemanick A., Haber-Sage S., Arbaiza R., Black M.M. (2019). CHAMP: A cluster randomized-control trial to prevent obesity in child care centers. Contemp. Clin. Trials.

[20] Hager E.R., Treuth M.S., Gormely C., Epps L., Snitker S., Black M.M. (2015). Ankle Accelerometry for Assessing Physical Activity Among Adolescent Girls: Threshold Determination, Validity, Reliability, and Feasibility. Res. Q. Exerc. Sport.

[21] Hager E.R., Gormley C.E., Latta L.W., Treuth M.S., Caulfield L.E., Black M.M. (2016). Toddler physical activity study: Laboratory and community studies to evaluate accelerometer validity and correlates. BMC Public Health.

[22] Slaton A., Kowalski A.J., Zemanick A., Kuhn A.P., Hager E.R., Black M.M. (2020). Motor Competence and Attainment of Global Physical Activity Guidelines among a Statewide Sample of Preschoolers. Int. J. Environ. Res. Public Health.

[23] Centers for Disease Control and Prevention Healthy Weight, Nutrition, and Physical Activity: Body Mass Index (BMI). [Cited 29 July 2021]. https://www.cdc.gov/healthyweight/assessing/bmi/index.html.

[24] Meyers L., Gamst G., Guarino A.J. (2017). Applied Multivariate Research: Design and Interpretation.

[25] Tandon P.S., Saelens B.E., Christakis D.A. (2015). Active Play Opportunities at Child Care. Pediatrics.

[26] Chim H., Gijselaers H.J., de Groot R.H., Van Gerven P.W., Egbrink M.G.O., Savelberg H.H. (2021). The effects of light physical activity on learning in adolescents: A systematic review. Int. Rev. Sport Exerc. Psychol.

[27] Ng J.Y.Y., He Q., Chong K.H., Okely A.D., Chan C.H.S., Ha A.S. (2021). The Impact of COVID-19 on Preschool-Aged Children’s Movement Behaviors in Hong Kong: A Longitudinal Analysis of Accelerometer-Measured Data. Int. J. Environ. Res. Public Health.

[28] Schmidt S.C.E., Anedda B., Burchartz A., Eichsteller A., Kolb S., Nigg C., Niessner C., Oriwol D., Worth A., Woll A. (2020). Physical activity and screen time of children and adolescents before and during the COVID-19 lockdown in Germany: A natural experiment. Sci. Rep..

[29] Fairclough S.J., Boddy L., Mackintosh K., Valencia-Peris A., Ramírez-Rico E. (2015). Weekday and weekend sedentary time and physical activity in differentially active children. J. Sci. Med. Sport.

[30] Azevedo M.R., Araújo C.L., da Silva M.C., Hallal P.C. (2007). Tracking of physical activity from adolescence to adulthood: A population-based study. Rev. Saúde Pública.

